# Direct comparison between surface imaging and orthogonal radiographic imaging for SRS localization in phantom

**DOI:** 10.1002/acm2.12498

**Published:** 2018-12-12

**Authors:** David Wiant, Han Liu, T. Lane Hayes, Qingyang Shang, Sasa Mutic, Benjamin Sintay

**Affiliations:** ^1^ Department of Radiation Oncology Cone Health Cancer Center Greensboro North Carolina 27403 USA; ^2^ Department of Radiation Oncology Washington University School of Medicine St. Louis Michigan 63110 USA

**Keywords:** intrafraction monitoring, SRS, surface imaging

## Abstract

**Purpose:**

Surface imaging (SI) offers a nonionizing, near real time alternative to radiographic imaging for intrafraction radiosurgery localization. In this work, we systematically compared a commercial SI system vs a commercial room mounted x‐ray localization system in phantom.

**Methods:**

An anthropomorphic head phantom with fiducial markers was imaged with linear accelerator on‐board x‐ray imaging, SI, and room mounted x‐ray imaging (RM) at ±45° and ±90° couch angles for three different head tilts and six different isocenters (72 total positions). The shifts generated by the three systems were compared as functions of couch angle, head tilt, and isocenter position with the on‐board imaging shifts used as ground truth. Two sample Kolmogorov–Smirnov tests were used to evaluate equivalence of the groups.

**Results:**

The magnitude of the displacement vectors for RM minus on‐board imaging and SI minus on‐board imaging over all 72 phantom positions were 0.7 ± 0.3 mm for both cases. The RM and SI showed no significant difference based on couch angle or isocenter position. Both systems showed decreasing accuracy with increasing couch angle, but both systems agreed with ground truth to <=1.1 mm at all couch angles. The exaggerated chin‐up head orientation showed significantly different shifts for SI and RM based on increased variance in the SI measurements, although both had submillimeter accuracy on average. The standard deviation of the real time SI displacement vector was <0.06 mm over all measurements, during which the on‐board imaging panels partially blocked the lateral camera pods for half the time.

**Conclusions:**

RM and SI showed similar accuracy over measurements at 72 different phantom positions. SI showed minimal performance loss with camera pods blocked. SI is a feasible option for intra‐fraction radiosurgery localization based on these phantom measurements.

## 1 INTRODUCTION

Stereotactic radiosurgery (SRS) has become a popular tool to treat intracranial brain metastases due to durable local control, convenience for the patients, and the possibility of reduced cognitive impairment vs whole brain radiotherapy.^1^, ^2^ One of the main technical recommendations for SRS is that patients can be localized to <1 mm during treatment.^3^ Accurate localization is accomplished with a combination of immobilization devices to limit patient movement and imaging to drive the patient to the correct position.

Intracranial SRS immobilization has typically been achieved with invasive head frames or thermoplastic masks. Both head frames^4^ and masks^5^ have been shown to limit intrafraction motion to about 1 mm. Localization during treatment to <1 mm has been achieved using immobilization devices with stereotactic coordinate systems and/or radiographic imaging.

Masks and radiographic imaging are a common localization method for linear accelerator (linac) based SRS. In many cases linac SRS will use multiple couch positions to create desirable dose distributions. Imaging systems native to conventional C‐arm linacs have limited ability to acquire orthogonal images at nonzero couch angles due to collisions with the patient/couch. Room mounted orthogonal x‐ray systems have been developed to enable intrafraction imaging at nonzero couch positions. These systems are considered independent of the linac. One such system is ExacTrac (ET) (BrainLAB AG, Feldkirchen, Germany).

Briefly, ET consists of two x‐ray tubes recessed in the floor and corresponding image receptors mounted on the ceiling that allow for nearly orthogonal images to be acquired at all couch positions. A 2D‐3D image registration is used to determine translations and rotations to align the patient with the reference image set. A typical workflow with ET has the patient initially positioned and verified with ET at zero couch position, this process is then repeated each time the couch is moved.^6^, ^7^

While ET has a proven clinical utility it does have some limitations, chiefly the (a) inability to provide real time patient monitoring, (b) imaging radiation dose, and (c) time required to review the image registrations. Non‐ionizing surface imaging (SI) is an alternative to radiographic imaging that does not have the limitations described above.

The AlignRT (ART) (Vision RT, London, UK) SI system consists of three ceiling mounted pods, each containing two cameras, that are used to generate a 3D surface at a rate of 2–6 frames/s. The camera generated surface is compared to a reference surface created based on contours from the patient's treatment plan or a surface from a prior ART image. The comparison is performed using a proprietary algorithm and results in translations and rotations to align the patient with the reference surface. Phantom studies at couch angles <90° have shown adequate positioning accuracy, however, there is limited data validating the use of ART at nonzero couch angles and when cameras are occluded.^8^, ^9^, ^10^, ^11^ Recently, Vision RT has developed a new optical setup technique, advanced camera optimization (ACO) that will be used in this work. Advanced camera optimization generates a 3‐dimensional optical calibration model for ART that is designed to provide enhanced tracking stability and accuracy over a wide range of treatment configurations.

Intrafraction motion measurement at nonzero couch angles is a hard task for imaging systems. Some of the key reasons are the difficulty in aligning the imaging isocenter to the linac isocenter and the wide array of possible patient configurations seen in the clinic. Misalignment of the imaging and linac isocenters is often not noticeable at zero couch angle. However, any misalignment will introduce errors in the image registration that increase in magnitude with increasing couch angle, negatively impacting patient localization. Different target positions and head configurations can lead to cases where minimal volume (or surface area) is available to the imaging system, which can limit the accuracy of image registrations. The couch positions and head orientations that yield reduced alignment information depend on the location of image receptors or cameras in the room. Both ET and ART are impacted by issues such as these in different ways. In this work, we directly compare ET and ART with ACO in a phantom over multiple couch angles and head orientations to evaluate the impact of couch rotation on each system.

## 2 MATERIALS AND METHODS

An ET dual generator system version 6.2.0 and an ART system version 5.0.1749 that underwent ACO calibration were evaluated in this study.

**Figure 1:**
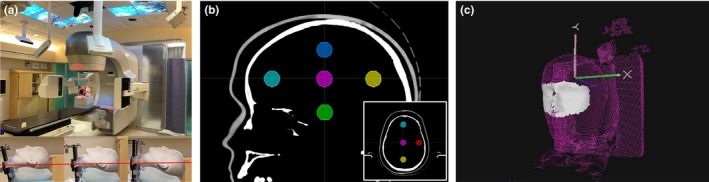
(a) The phantom setup with imaging arms extended. The inset shows close‐up side views of the phantom in chin‐down, neutral, and chin‐up orientations. The red line is a reference for head tilt. (b) Sagittal view of the phantom showing the superior, inferior, center, anterior, and posterior isocenter positions. The inset has an axial view showing the left isocenter. (c) AlignRT region of interest.

An anthropomorphic head phantom with multiple 3 mm diameter titanium spherical fiducial markers was scanned in three different orientations (exaggerated 8° chin‐up, 8° chin‐down, neutral) on a Philips computed tomography scanner (Brilliance Big Bore, Philips, Cleveland, OH) with a 1 mm slice thickness and fields of view large enough to cover the phantom. The scans were sent to a commercial treatment planning system (Eclipse, Varian, Palo Alto, CA) where a body contour and six different targets were created. Plans with isocenters at the target centers and fields at 0°, ±45°, and ±90° couch positions were created on each scan. The 18 treatment plans with unique treatment orientations were exported in DICOM format to ART and ET (Fig. 1).

The intracranial SRS site was used in ART for each plan, with a region of interest (ROI) that extended from the supraorbital ridge to the inferior edge of the nose in the superior–inferior dimension and to the midpoints between the eyes and the ears in the lateral dimension (Fig. 1). This ROI was selected because it was felt to be the smallest ROI that would be used in a clinical setting. In ET, the midlevel intracranial settings (80 kV, 6.3 mA) were selected.

All measurements were made on a Varian TrueBeam STx (TB) C‐arm linac. For each isocenter the phantom was initially setup at couch = 0° using ET with residual shifts <0.5 mm and 0.5°. The residual ET shifts were recorded. At this point a new ART reference surface was acquired. Then an orthogonal image pair (anterior–posterior 2.5 MV image, lateral variable energy KV image) was acquired using the TB on‐board imaging system. The ART cameras were on for >15 min prior to testing to allow for thermal stabilization.

At ±45° and ±90° couch positions ET images and orthogonal TB images were acquired. The ART system was left running the entire time with the real time shifts recorded in a text file. Note, the phantom was setup such that the TB imaging arms could be extended at all couch positions without moving the gantry or phantom (Fig. 1).

Offline, the residual ET shifts at couch = 0° were subtracted from the ET shifts at each couch position to get ET displacements. The ART shifts at each couch angle were extracted from the text file using a custom Matlab (Mathworks, Natick, MA) program that found the average and standard deviations of the shifts. The TB images were also analyzed in a custom Matlab program that identified the center of the fiducial markers on each image pair and used these points to calculate the optimal translations and rotations from the initial couch = 0° images. Briefly, the transforms were found by (a) identifying the centroids of each point group, (b) moving the centroid of each point group to the origin, (c) determining the optimal rotation using singular value decomposition, (d) applying the rotation to the target centroid, and (e) finding the difference between the rotated centroid and the reference centroid (to get the translations).^12^

The equivalence of the ART and ET shifts were compared to each other and to the TB shifts as a function of couch position, isocenter position, and phantom orientation using two sample Kolmogorov–Smirnov (KS) tests.

## 3 RESULTS

The TB measurements will be considered the “ground truth” in the following evaluations. The main sources of uncertainty in these measurements were the (a) KV image panel positioning errors/hysteresis over repeated movements (the MV panel remained stationary) and (b) fiducial marker center identification. The KV image panel variance was evaluated by extending the panel from mid to full extension five consecutive times with an image acquired at each cycle. Fiducial markers were identified on each image. Analysis of the marker positions showed a variance of ±0.1 mm in each dimension over the five cycles. The fiducial marker center identification was a semiautomated process, where the user defined the region of the marker and the software identified the center of the marker. The smooth, spherical shape of the markers allowed for faithful interpolation of the images from a voxel size of 0.388 to 0.065 mm. Repeated tests of marker detection gave uncertainty on the order 0.1 mm. Based on these findings the total uncertainty of the marker positions was on the order of 0.1–0.2 mm per image, which gives an uncertainty of about 0.3 mm for the displacement between images.

**Figure 2:**
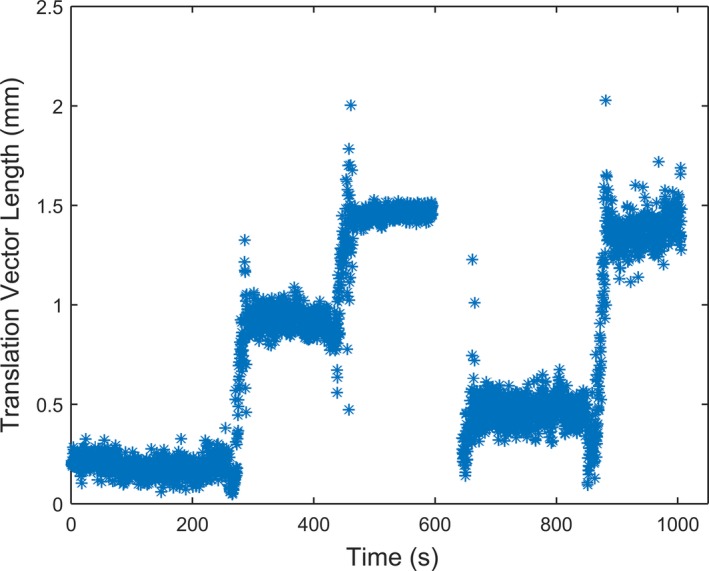
The real time AlignRT displacement vector magnitude for a representative phantom orientation. The steps represent couch motion. The flat regions represent couch = 0°, 45°, 90°, −45°, 90° from left to right. Within each flat region the imaging arms were extended for half of the time and retracted for the remaining time.

The ART shifts were collected continuously for each isocenter position. Figure 2 shows the magnitude of the displacement vector at one position. The steps in the plot represent couch movement. In between each step the TB KV image panels were extended and retracted to acquire images, partially blocking the lateral cameras when extended (Fig. 1). The shifts were recorded the entire time between steps, i.e., with image panels extended and retracted. The mean SD's for all translations and rotations over all couch positions were <0.06 mm. The maximum SD for any couch position was <0.16 mm.

The ET registration uncertainty was evaluated by repeating the image registration process without acquiring new images on five image sets. Typical changes for the shifts were on the order of 0.1–0.2 mm and degrees.

**Figure 3:**
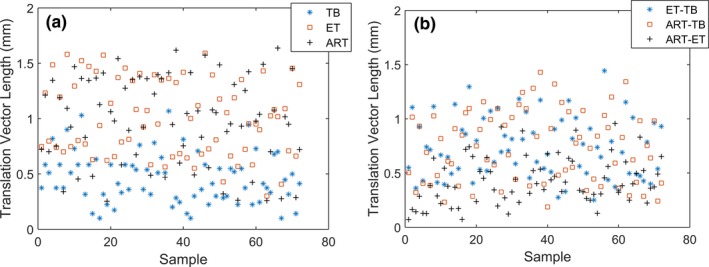
(a) The displacement vector magnitudes for all ExacTrac, AlignRT, and TrueBeam measurements. (b) The displacement vector magnitudes between ExacTrac‐TrueBeam, AlignRT‐TrueBeam, and AlignRT‐ExacTrac.

The displacement vector magnitudes for all measurements and the displacement vectors between ET‐TB, ART‐TB, and ART‐ET are shown in Fig. 3. The means of the differences in ET‐TB, ART‐TB, and ART‐ET were 0.7 ± 0.3 mm (0.3–1.4 mm), 0.7 ± 0.3 mm (0.2–1.4 mm), and 0.4 ± 0.2 mm (0.1–1.0 mm) respectively (±1 SD, range). The KS tests showed that the ART and ET displacements were equivalent (*P* = 0.46) and the ART‐TB and ET‐TB displacements were equivalent (*P* = 0.60).

**Figure 4:**
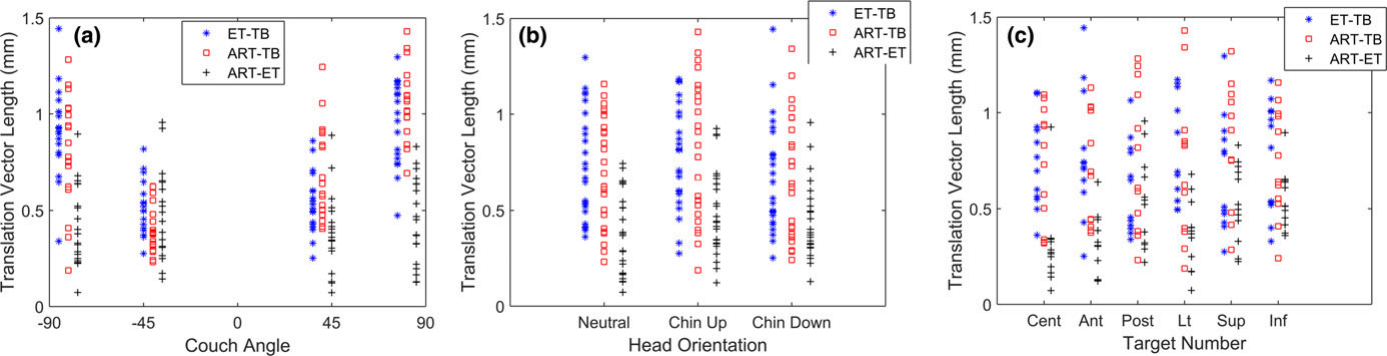
The displacement magnitudes between ExacTrac‐TrueBeam, AlignRT‐TrueBeam, and AlignRT‐ExacTrac as a function of (a) couch angle (b) phantom orientation, and (c) isocenter position.

Translations and rotations for all positions are shown as functions of phantom orientation, isocenter, and couch position in Table 1. The displacement magnitudes as functions of phantom orientation, isocenter, and couch position are plotted in Fig. 4. The KS tests showed that all groupings of the displacement magnitudes for ET‐TB and ART‐TB are similar (*P* >= 0.10) except for the phantom chin‐up orientation (*P* = 0.01).

**Table 1:** Differences, translations, and rotations as functions of couch angle, phantom orientation, and isocenter position. ART = AlignRT, ET = ExacTrac, TB = TrueBeam

	Magnitude (mm)	Vertical (mm)	Longitudinal (mm)	Lateral (mm)	Yaw (°)	Roll (°)	Pitch (°)
ART‐TB: all data	0.7 ± 0.3 (0.2–1.4)	0.2 ± 0.2 (0.0–0.6)	0.3 ± 0.2 (0.0–1.1)	0.5 ± 0.3 (0.0–1.4)	0.3 ± 0.3 (0.0–1.3)	0.2 ± 0.1 (0.0–0.7)	0.3 ± 0.2 (0.0–0.9)
ET‐TB: all data	0.7 ± 0.3 (0.3–1.4)	0.2 ± 0.2 (0.0–0.7)	0.4 ± 0.3 (0.0–1.1)	0.5 ± 0.3 (0.0–1.1)	0.4 ± 0.4 (0.0–1.6)	0.2 ± 0.2 (0.0–0.8)	0.3 ± 0.2 (0.0–0.9)
ART‐ET: all data	0.4 ± 0.2 (0.1–1.0)	0.2 ± 0.2 (0.0–0.8)	0.3 ± 0.2 (0.0–1.0)	0.2 ± 0.1 (0.0–0.6)	0.2 ± 0.1 (0.0–0.7)	0.2 ± 0.1 (0.0–0.7)	0.3 ± 0.2 (0.0–0.8)
ART‐TB: neutral head	0.7 ± 0.3 (0.2–1.2)	0.2 ± 0.2 (0.0–0.6)	0.3 ± 0.2 (0.0–0.7)	0.5 ± 0.3 (0.1–1.1)	0.3 ± 0.3 (0.0–1.0)	0.1 ± 0.1 (0.0–0.4)	0.3 ± 0.2 (0.1–0.5)
ET‐TB: neutral head	0.7 ± 0.3 (0.4–1.3)	0.2 ± 0.2 (0.0–0.7)	0.4 ± 0.3 (0.0–0.9)	0.5 ± 0.3 (0.0–1.1)	0.4 ± 0.4 (0.0–1.1)	0.1 ± 0.1 (0.0–0.6)	0.3 ± 0.2 (0.1–0.8)
ART‐ET: neutral head	0.4 ± 0.2 (0.1–0.7)	0.2 ± 0.2 (0.0–0.7)	0.2 ± 0.2 (0.0–0.6)	0.1 ± 0.1 (0.0–0.3)	0.2 ± 0.1 (0.0–0.4)	0.2 ± 0.2 (0.0–0.7)	0.2 ± 0.2 (0.0–0.6)
ART‐TB: chin up	0.8 ± 0.4 (0.2–1.4)	0.3 ± 0.2 (0.0–0.6)	0.3 ± 0.3 (0.0–1.1)	0.6 ± 0.4 (0.0–1.4)	0.4 ± 0.4 (0.0–1.3)	0.2 ± 0.2 (0.0–0.7)	0.4 ± 0.2 (0.0–0.9)
ET‐TB: chin up	0.8 ± 0.3 (0.3–1.2)	0.2 ± 0.2 (0.0–0.7)	0.4 ± 0.3 (0.0–1.0)	0.5 ± 0.3 (0.1–1.1)	0.5 ± 0.5 (0.0–1.6)	0.2 ± 0.2 (0.0–0.8)	0.4 ± 0.2 (0.0–0.9)
ART‐ET: chin up	0.5 ± 0.2 (0.1–0.9)	0.2 ± 0.2 (0.1–0.8)	0.3 ± 0.2 (0.0–0.8)	0.2 ± 0.1 (0.0–0.5)	0.3 ± 0.2 (0.0–0.6)	0.2 ± 0.1 (0.0–0.4)	0.3 ± 0.2 (0.0–0.8)
ART‐TB: chin down	0.7 ± 0.3 (0.2–1.3)	0.2 ± 0.1 (0.0–0.6)	0.4 ± 0.2 (0.0–0.8)	0.5 ± 0.3 (0.0–1.1)	0.3 ± 0.3 (0.0–1.2)	0.1 ± 0.1 (0.0–0.3)	0.2 ± 0.2 (0.0–0.5)
ET‐TB: chin down	0.7 ± 0.3 (0.3–1.4)	0.2 ± 0.2 (0.0–0.7)	0.4 ± 0.3 (0.0–1.1)	0.4 ± 0.3 (0.0–1.1)	0.4 ± 0.4 (0.0–1.6)	0.2 ± 0.1 (0.0–0.5)	0.3 ± 0.1 (0.1–0.6)
ART‐ET: chin down	0.4 ± 0.2 (0.1–1.0)	0.1 ± 0.1 (0.0–0.5)	0.3 ± 0.2 (0.0–1.0)	0.2 ± 0.1 (0.0–0.7)	0.2 ± 0.1 (0.0–0.7)	0.2 ± 0.1 (0.0–0.5)	0.3 ± 0.2 (0.0–0.6)
ART‐TB: table = 45°	0.7 ± 0.2 (0.4–1.2)	0.1 ± 0.1 (0.0–0.5)	0.4 ± 0.3 (0.0–1.1)	0.5 ± 0.2 (0.3–0.8)	0.2 ± 0.2 (0.0–0.5)	0.1 ± 0.2 (0.0–0.7)	0.3 ± 0.1 (0.1–0.4)
ET‐TB: table = 45°	0.5 ± 0.2 (0.3–0.9)	0.1 ± 0.2 (0.0–0.5)	0.2 ± 0.1 (0.0–0.6)	0.4 ± 0.1 (0.1–0.7)	0.2 ± 0.1 (0.0–0.4)	0.2 ± 0.2 (0.0–0.5)	0.2 ± 0.1 (0.0–0.4)
ART‐ET: table = 45°	0.4 ± 0.2 (0.1–0.9)	0.2 ± 0.2 (0.0–0.6)	0.3 ± 0.2 (0.1–0.8)	0.1 ± 0.1 (0.0–0.4)	0.2 ± 0.1 (0.0–0.5)	0.2 ± 0.1 (0.0–0.5)	0.2 ± 0.2 (0.0–0.6)
ART‐TB: table = 90°	1.1 ± 0.2 (0.7–1.4)	0.3 ± 0.2 (0.0–0.6)	0.3 ± 0.2 (0.1–0.8)	0.9 ± 0.2 (0.7–1.4)	0.7 ± 0.3 (0.3–1.3)	0.1 ± 0.1 (0.0–0.2)	0.4 ± 0.2 (0.0–0.6)
ET‐TB: table = 90°	0.9 ± 0.2 (0.5–1.3)	0.3 ± 0.2 (0.0–0.7)	0.3 ± 0.2 (0.0–0.6)	0.8 ± 0.3 (0.2–1.1)	0.9 ± 0.4 (0.1–1.6)	0.1 ± 0.2 (0.0–0.6)	0.4 ± 0.2 (0.1–0.8)
ART‐ET: table = 90°	0.4 ± 0.2 (0.1–0.8)	0.2 ± 0.2 (0.0–0.7)	0.3 ± 0.2 (0.0–0.6)	0.2 ± 0.2 (0.0–0.7)	0.3 ± 0.2 (0.0–0.7)	0.2 ± 0.2 (0.0–0.7)	0.1 ± 0.1 (0.0–0.4)
ART‐TB: table = −45°	0.4 ± 0.1 (0.2–0.6)	0.2 ± 0.2 (0.0–0.6)	0.2 ± 0.1 (0.0–0.6)	0.2 ± 0.1 (0.0–0.3)	0.2 ± 0.2 (0.0–0.7)	0.2 ± 0.1 (0.0–0.5)	0.2 ± 0.2 (0.0–0.9)
ET‐TB: table = −45°	0.5 ± 0.1 (0.3–0.8)	0.2 ± 0.2 (0.0–0.6)	0.4 ± 0.1 (0.0–0.7)	0.2 ± 0.1 (0.0–0.4)	0.2 ± 0.2 (0.0–0.8)	0.2 ± 0.2 (0.0–0.8)	0.3 ± 0.2 (0.1–0.7)
ART‐ET: table = −45°	0.5 ± 0.2 (0.1–1.0)	0.2 ± 0.2 (0.0–0.8)	0.3 ± 0.2 (0.0–1.0)	0.1 ± 0.1 (0.0–0.5)	0.2 ± 0.1 (0.0–0.5)	0.2 ± 0.1 (0.0–0.4)	0.3 ± 0.2 (0.0–0.6)
ART‐TB: table = −90°	0.8 ± 0.3 (0.2–1.3)	0.3 ± 0.2 (0.0–0.6)	0.5 ± 0.2 (0.1–0.8)	0.5 ± 0.3 (0.1–1.1)	0.3 ± 0.3 (0.0–1.1)	0.2 ± 0.1 (0.1–0.3)	0.3 ± 0.2 (0.1–0.7)
ET‐TB: table = −90°	0.9 ± 0.2 (0.3–1.4)	0.2 ± 0.1 (0.0–0.4)	0.7 ± 0.2 (0.3–1.1)	0.5 ± 0.2 (0.1–1.0)	0.5 ± 0.4 (0.1–1.4)	0.1 ± 0.1 (0.0–0.3)	0.3 ± 0.2 (0.1–0.9)
ART‐ET: table = −90°	0.4 ± 0.2 (0.1–0.9)	0.2 ± 0.1 (0.0–0.5)	0.3 ± 0.2 (0.0–0.7)	0.2 ± 0.1 (0.0–0.5)	0.3 ± 0.1 (0.1–0.6)	0.2 ± 0.2 (0.0–0.7)	0.5 ± 0.2 (0.2–0.8)
ART‐TB: target center	0.7 ± 0.3 (0.3–1.1)	0.2 ± 0.1 (0.0–0.5)	0.4 ± 0.2 (0.1–0.6)	0.5 ± 0.3 (0.0–0.9)	0.2 ± 0.2 (0.0–0.7)	0.1 ± 0.1 (0.0–0.2)	0.2 ± 0.1 (0.0–0.4)
ET‐TB: target center	0.7 ± 0.2 (0.4–1.1)	0.2 ± 0.2 (0.0–0.7)	0.5 ± 0.2 (0.1–0.8)	0.5 ± 0.2 (0.2–0.9)	0.3 ± 0.3 (0.0–1.1)	0.1 ± 0.1 (0.0–0.2)	0.3 ± 0.1 (0.0–0.5)
ART‐ET: target center	0.3 ± 0.2 (0.1–0.9)	0.2 ± 0.2 (0.0–0.8)	0.2 ± 0.1 (0.0–0.4)	0.1 ± 0.1 (0.0–0.3)	0.3 ± 0.1 (0.0–0.6)	0.1 ± 0.1 (0.0–0.4)	0.3 ± 0.2 (0.0–0.7)
ART‐TB: target anterior	0.7 ± 0.3 (0.4–1.1)	0.2 ± 0.2 (0.0–0.5)	0.2 ± 0.2 (0.0–0.7)	0.6 ± 0.3 (0.2–0.9)	0.7 ± 0.3 (0.3–1.1)	0.2 ± 0.1 (0.0–0.5)	0.2 ± 0.1 (0.1–0.4)
ET‐TB: target anterior	0.8 ± 0.3 (0.3–1.4)	0.3 ± 0.2 (0.0––0.6)	0.4 ± 0.3 (0.0–1.1)	0.6 ± 0.3 (0.1–1.0)	0.8 ± 0.5 (0.1–1.6)	0.2 ± 0.2 (0.0–0.8)	0.4 ± 0.3 (0.1–0.9)
ART‐ET: target anterior	0.3 ± 0.2 (0.1–0.6)	0.1 ± 0.1 (0.0–0.3)	0.2 ± 0.2 (0.0–0.6)	0.1 ± 0.1 (0.0–0.4)	0.2 ± 0.1 (0.0–0.5)	0.2 ± 0.1 (0.0–0.4)	0.2 ± 0.2 (0.0–0.7)
ART‐TB: target posterior	0.8 ± 0.4 (0.2–1.3)	0.3 ± 0.2 (0.1–0.6)	0.5 ± 0.3 (0.1–1.1)	0.5 ± 0.3 (0.1–1.0)	0.5 ± 0.4 (0.0–1.3)	0.2 ± 0.2 (0.0–0.7)	0.4 ± 0.3 (0.0–0.9)
ET‐TB: target posterior	0.6 ± 0.2 (0.3–1.1)	0.3 ± 0.2 (0.0–0.7)	0.3 ± 0.2 (0.0–0.6)	0.3 ± 0.3 (0.0–0.8)	0.6 ± 0.5 (0.0–1.6)	0.2 ± 0.1 (0.0–0.5)	0.3 ± 0.3 (0.0–0.7)
ART‐ET: target posterior	0.5 ± 0.2 (0.2–1.0)	0.1 ± 0.1 (0.0–0.3)	0.4 ± 0.3 (0.0–1.0)	0.2 ± 0.1 (0.1–0.5)	0.3 ± 0.1 (0.1–0.5)	0.1 ± 0.1 (0.0–0.4)	0.3 ± 0.2 (0.0–0.5)
ART‐TB: target left	0.7 ± 0.4 (0.2–1.4)	0.1 ± 0.1 (0.0–0.2)	0.4 ± 0.2 (0.1–0.8)	0.5 ± 0.4 (0.1–1.4)	0.2 ± 0.2 (0.0–0.6)	0.1 ± 0.1 (0.0–0.3)	0.3 ± 0.2 (0.0–0.6)
ET‐TB: target left	0.8 ± 0.3 (0.5–1.2)	0.1 ± 0.1 (0.0––0.3)	0.5 ± 0.3 (0.1–0.9)	0.5 ± 0.3 (0.1–1.1)	0.3 ± 0.2 (0.0–0.8)	0.1 ± 0.1 (0.0–0.3)	0.3 ± 0.1 (0.1–0.5)
ART‐ET: target left	0.4 ± 0.2 (0.7–0.1)	0.1 ± 0.1 (0.0–0.4)	0.3 ± 0.2 (0.1–0.7)	0.1 ± 0.1 (0.0–0.3)	0.2 ± 0.1 (0.0–0.3)	0.2 ± 0.1 (0.0–0.3)	0.3 ± 0.2 (0.1–0.8)
ART‐TB: target superior	0.8 ± 0.3 (0.3–1.3)	0.2 ± 0.1 (0.1–0.4)	0.3 ± 0.2 (0.1–0.8)	0.6 ± 0.4 (0.1–1.3)	0.2 ± 0.2 (0.1–0.6)	0.1 ± 0.1 (0.0–0.3)	0.2 ± 0.2 (0.0–0.5)
ET‐TB: target superior	0.7 ± 0.3 (0.3–1.3)	0.3 ± 0.2 (0.0–0.7)	0.3 ± 0.2 (0.1–0.7)	0.5 ± 0.3 (0.1–1.1)	0.4 ± 0.4 (0.1–1.2)	0.3 ± 0.2 (0.0–0.6)	0.3 ± 0.2 (0.1–0.8)
ART‐ET: target superior	0.5 ± 0.2 (0.2–0.8)	0.3 ± 0.2 (0.0–0.7)	0.2 ± 0.1 (0.0–0.4)	0.2 ± 0.2 (0.0–0.7)	0.3 ± 0.2 (0.1–0.7)	0.4 ± 0.2 (0.0–0.7)	0.3 ± 0.2 (0.0–0.7)
ART‐TB: target inferior	0.7 ± 0.3 (0.2–1.2)	0.4 ± 0.2 (0.1–0.6)	0.3 ± 0.2 (0.0–0.7)	0.5 ± 0.4 (0.0–1.1)	0.2 ± 0.2 (0.0–0.5)	0.1 ± 0.1 (0.0–0.3)	0.3 ± 0.1 (0.1–0.6)
ET‐TB: target inferior	0.8 ± 0.3 (0.3–1.2)	0.1 ± 0.1 (0.0–0.3)	0.5 ± 0.4 (0.0–1.0)	0.5 ± 0.3 (0.1–1.1)	0.3 ± 0.2 (0.0–0.7)	0.1 ± 0.1 (0.0–0.3)	0.3 ± 0.2 (0.1–0.6)
ART‐ET: target inferior	0.6 ± 0.1 (0.4–0.9)	0.3 ± 0.1 (0.0–0.5)	0.4 ± 0.1 (0.2–0.6)	0.1 ± 0.1 (0.0–0.5)	0.2 ± 0.1 (0.0–0.4)	0.1 ± 0.1 (0.0–0.3)	0.3 ± 0.2 (0.0–0.6)

## 4 DISCUSSION

The TB displacement magnitudes from couch walkout ranged from 0.1 to 1.1 mm, with the largest displacements at −90° couch position. These measurements are in good agreement with more than 5 yr of Winston‐Lutz measurements on this machine that show up to 0.9 mm displacement at −90° couch position. These results are also in good agreement with a multi‐institution Winston‐Lutz study that showed 0.5–0.7 mm isocenter displacement over five TB's.^13^ The TB measurements appear to be a reasonable ground truth for comparisons.

The ET displacement vector magnitudes showed an average difference of 0.7 mm vs TB (maximum difference of 1.4 mm) for all measurements. Previous phantom studies found ET positional accuracy to range from 0.6 to 1.25 mm for measurements at 0° couch with up to 4° of phantom rotation.^14^, ^15^, ^16^ Feygelman et al.^17^ used ET to localize a skull phantom (residual shifts < 0.3 mm, <0.3°) for multiple hidden target tests that covered six couch angles. They found ET positional accuracy of 0.83 mm (range 0.33–1.46 mm). Our measurements are in excellent agreement with these reports, which indicates that our ET system is representative of a typical practice.

The ART displacement vector magnitudes showed an average difference of 0.7 mm vs TB (maximum difference of 1.4 mm) for all measurements. Oliver et al.^8^ used ART to perform hidden target tests with a head phantom at couch angles of 315°, 330°, and 345°, which yielded a localization accuracy of 0.9 mm. Li et al.^9^ reported a 0.5 mm positional accuracy in phantom at ±90° couch, found by subtracting independently measured couch “walkout” from reported ART shifts. Cervino et al.^10^ compared ART to the Varian Optical Guidance Platform (OGP) at couch angles from −90° to 90° and found differences between the two systems of 0.5 to 1.1 mm (This work cited a localization accuracy of 1.1 mm for OGP). Peng et al. also performed comparisons between ART and OGP. In this work, phantom translations and rotations were introduced over −90° to 90° couch angles. Peng and colleagues found a mean agreement of 1.2 mm between the systems with a maximum difference of 2.3 mm. The ART phantom measurements discussed above are in reasonable agreement with this work, again indicating that our system and calibration are representative.

Up to this time, the use of ART to guide SRS localization without intrafraction radiographic images has been limited due to minimal data evaluating the system's stability and accuracy at nonzero couch angles. Mancosu et al.^18^ showed that ART was able to track known phantom displacements up to 3 cm with submillimeter accuracy at 0°, 45°, and 90° couch. However, they examined shifts at each couch position independently and did not include the impact of couch rotation. Cervino's and Peng's work reported that ART positional accuracy was worst at the ±90° couch angles. However, no attempt has been made to systematically study the impact of rotation on ART and to place it in context with current technologies.

We found that ART and ET showed comparable positional accuracy at all couch angles, and that both systems showed decreasing accuracy with increasing couch angle. This indicates that misalignment of the ET and ART isocenters with the couch rotation center is likely a driving factor in decreased accuracy at nonzero couch angles. The displacement of a point after rotation due to isocenter misalignment is given by:

(1) $$Offset\;Magnitude=\sqrt{dz^{2}+\left(dx^{2}+dy^{2}\right)\left({\left[\cos\varphi -1\right]}^{2}+\sin^{2}\varphi \right)}$$

where φ = couch angle, *dx*,* dy*, and *dz* are isocenter misalignments in the left–right, superior–inferior, and anterior–posterior dimensions respectively. The impact of isocenter misalignment increases with increasing couch angle. For example a 0.2 mm misalignment, which is quite reasonable, gives 0.3 mm error at ±90°.

The chin‐up orientation was the only measurement group to show a significant difference between ART and ET. The ET and ART had similar mean displacement magnitudes, but the ART measurements showed a wider spread. The chin‐up position directs the ART measurements ROI away from the cameras, decreasing the surface area available to image when the phantom superior–inferior axis is parallel to the sight line of the cameras. This essentially turned the 3 camera pod system into a 2 pod system, which appeared to slightly decrease accuracy. Although, this head tilt is likely larger than what is typically seen with patients and ART produced sub‐millimeter accuracy, head tilt should be an important consideration for patient setup.

The stability or noise of an imaging system is an important factor for treatments with tight tolerances. As the SD becomes >0.3 mm the chance that a measurement of patient in the correct position will lead to a result >1.0 mm greatly increases (i.e., a 1.0 mm result falls within 3 SD for a measurement with a mean value of 0 mm). Mancosu et al.^18^ reported SD up to 0.8 mm when measurements were made with at gantry = 45° and couch = 0°. Peng et al. showed that blocking a camera POD resulted in ART shift changes up to 0.4 mm. In this work, we found a mean ART SD of 0.06 mm (maximum of 0.16 mm), which included measurements with both TB imaging arms extended partially blocking the lateral cameras. In the worst case, camera blockage changed the ART shifts < 0.2 mm. Partial blockage of 2 pods is not identical to complete blockage of a single pod as in the other works, however, it reduces the ROI area available to the system and seems to indicate improved noise and stability after ACO calibration.

It is worth mentioning that ART gave displacement magnitudes up to 0.2 mm at couch = 0° immediately after a new reference surface was acquired. These offsets were felt to be errors in the surface registrations likely resulting from low levels of noise. These offsets were included in the displacement results at nonzero couch positions because the authors considered it relevant to the systems accuracy. However, another option is to subtract the couch = 0° shift values from the nonzero couch values, which result in the reported ART measurements being about 0.2 mm closer to the TB measurements.

The ET stability/repeatability measurements in this work were limited. However, a more extensive review of ET stability over 50 registrations on the same image sets showed uncertainty in each dimension of 0.2–0.3 mm,^19^ which is slightly larger than the small sampling in this work. Based on this data, ART measurements in this work show comparable or better stability than ET.

The ET and ART measurements were much closer to each other than to the TB measurements. This points to a systematic error in the radiographic calibration of the two systems. Both systems use a geometric phantom and TB MV images to match their isocenters to the TB mechanical isocenter. Any error in the TB MV image center relative to the TB mechanical isocenter would yield a rotationally dependent systematic error across both systems similar to what was described in Eq. (1). This would be additive to any error in the ET/ART to TB MV isocenter alignment.

This work shows near identical performance of ET and ART for SRS intrafraction localization. The main limitations are the narrow scope of the study. The ART system performance may vary with skin tone (as evidenced by the option to select skin tone in the application) and head shape. This work only examined a single skin tone and head shape, other configurations may yield different system performance. A single ROI size was used in this work, however, it is the smallest area that would typically be used for SRS localization. It is likely that any increase in ROI size would give comparable of better ART performance. Previous work showed that ET positional accuracy is dependent on x‐ray energy, which affects boney anatomy contrast.^19^ Only a single energy was used in this work.

## 5 CONCLUSIONS

Based purely on phantom measurements the current ART hardware with ACO calibration appears to be suitable for intrafraction SRS localization. It offers real time monitoring with accuracy comparable to ET based on phantom measurements. Isocenter calibration appears to be the driving factor for the accuracy of both systems, as such it should be an important consideration in SRS imaging. Variation in patient anatomy and orientation, along with motion of the brain relative to either the skull or the skin surface are important considerations in SRS imaging that cannot be tested in phantom. Next steps are to continue the ET to ART comparison in a prospective clinical trial to further explore these questions.

## CONFLICT OF INTEREST

This work received nonfinancial support from Vision RT. Dr. Mutic reports grants from ViewRay, Inc, grants and other from Varian Medical Systems, other from TreatSafely, LLC, other from Radialogica, LLC, outside the submitted work.
